# Medicine

**Published:** 1898-03

**Authors:** Theodore Fisher


### Progress of the Medical Sciences.

### MEDICINE.

During the past half-year **typhoid fever** has been brought prominently before the medical profession, and accounts of the origin and spread of epidemics, papers on Widal's reaction, and discussions upon treatment, may have filled the minds of medical men to satiation. There is, however, one feature of typhoid fever at which it may be pardonable to take a passing glance. That feature is **the liability of typhoid fever to give rise to complications** that, owing to their comparative infrequency, run the risk of being ignored. One can understand how such serious complications as intestinal perforation and hemorrhage may throw all others into the background, but one is perhaps hardly prepared to find it stated by a French writer that he finds no mention of lobar pneumonia as a complication of typhoid fever in French text-books, and in journals at his disposal only once[1]. Consolidation of the lungs and other complications may not have forced themselves under our notice, either by their frequency or by the sudden urgency of the symptoms they produce, yet it is important that we should recognise the possibility of their onset.

It was formerly thought that typhoid fever was almost entirely a local disease, and that the general symptoms were due to the absorption of toxins. This is no doubt in great measure true, but it is now known that not only toxins but various micro-organisms find their way in considerable numbers into the blood. The typhoid bacillus itself, although difficult to detect in the blood, obviously enters the circulation, since it can generally be found in the spleen and frequently in the urine. Not only does the typhoid bacillus enter the blood in ordinary cases of typhoid fever, but it may enter and cause death without having given rise to any intestinal lesion, as is shown by recently recorded cases by Cheadle,[2] Goodall,[3] Nicholls and Keenan,[4] and Beatty.[5]

Although not directly in accord with the subject, perhaps

[1] Auché et Carrière, *Arch. clin. de Bordeaux*, 1897, vi. 395.

[2] *Lancet*, 1897, ii. 254. [3] *Ibid.*, 1897, i. 523.

[4] *Montreal M. J.*, 1898, xxvii. 9. [5] *Tr. Roy. Acad. M. Ireland*, 1897, xv. 54.

it may be interesting, in connection with the ulceration or absence of ulceration of the intestines, to refer to a recent case that occurred in the *post-mortem* room of the Bristol Royal Infirmary. In this case the small intestine had almost entirely escaped, one small ulcer only being present; but the **ulceration of the large intestine** was extreme, and extended from the cæcum to the rectum. A few somewhat similar cases are recorded in the London Pathological Society's *Transactions;* it seems, therefore, that although in most people the Peyer's patches and solitary follicles of the small intestine are most vulnerable to the typhoid bacillus, in others they escape, while the solitary follicles of the large intestine suffer. In others again, although both the lymphatic structures of the small and large intestine can resist invasion, the bacillus is able to find entrance to the blood with a fatal result.

Before leaving the subject of ulceration of lymphoid structures, it may be mentioned that it apparently occasionally occurs in one other situation, that is at **the pyloric end of the stomach,** and may possibly be due to the typhoid bacillus or associated organisms attacking the lymphoid follicles there. Charlewood Turner [1] records a small shallow ulcer of the pylorus in typhoid fever, and two similar cases are described in the *post-mortem* records of the Bristol Royal Infirmary. In both of these cases two small ulcers were present at the pylorus, and of one it is stated that recent hemorrhage had apparently taken place from it. Cases of hæmatemesis in typhoid fever are recorded by Green and Galloway,[2] but in the one case of these that ended fatally no ulcer was found.

To return, however, to the presence of the typhoid bacillus, or of the organisms which generally accompany it, in the blood, it must be obvious that their escape into the circulation exposes the individual attacked to the risk of **pathological manifestations in various parts of the body.** Such manifestations may occur, and it is perhaps to be wondered at that they do not occur more often.

Micro-organisms being present in the blood, it is natural first to think of their effect upon **the blood itself.** Thrombosis is the first result that occurs to us; and thrombosis of the femoral vein we know may complicate typhoid fever, resulting in rare cases in pulmonary embolism.[3] In other cases the pulmonary artery may be blocked by a primary thrombosis.[4] A situation almost as serious for thrombosis is in the veins[5] or arteries of the brain giving rise to hemiplegia[6] or other manifestations[7] of a cerebral lesion. *Ante-mortem* clotting may also occur in one of the ventricles of the heart, especially dangerous on the left side on account of the

[1] *Tr. Path. Soc. Lond.*, 1884, xxxv. 208. [2] *Lancet*, 1895, ii. 1498.
[3] Fagge, *Tr. Path. Soc. Lond.*, 1876, xxvii. 70; Saw, *Med. Press & Circ.*, 1897, [cxv.] 453.
[4] Hunter, *Lancet*, 1896, ii. 1459.
[5] Richardson, *J. Nerv. & Ment. Dis.*, 1897, xxiv. 404.
[6] Donkin, *Lancet*, 1892, i. 912. [7] Osler, *Johns Hopkins Hosp. Rep.*, 1895, v. 469.

possibility of cerebral embolism.[1] Probably thrombosis of less serious nature, occurring in situations where it fails to give rise to symptoms, is not uncommon: for example, a division of the renal vein to one kidney was blocked by an *ante-mortem* clot in a recent case at the Bristol Royal Infirmary. Dr. Symes found the bacillus coli commune in this thrombus, and such limited clotting may be due to multiplication of bacilli at one particular spot. The blood, however, may suffer, not only locally but throughout the body. We are all familiar with the increasing pallor of typhoid patients, but may be unaware, as Thayer[2] remarks, "that a post-typhoid anæmia may in some instances become so profound as to be of itself a grave complication of the disease." After referring to previous literature on the subject, Thayer reports two cases—one in a man aged 30 years, another in a man aged 25 years. In the seventh week of the fever the hæmoglobin was about 26 per cent. of the normal in the first case; and below 20 per cent. in the fourth week of the second. Both cases recovered. While speaking of the blood, I may digress again for a moment to refer to a small point in connection with the spleen, an organ intimately associated with blood nutrition. In acute diseases in which micro-organisms find entrance to the blood, it is in the spleen the organisms are most readily found, and there is generally enlargement of that body. The spleen in typhoid fever is no exception to the rule, and to endeavour to detect it by palpation is one of the first impulses of the physician on seeing a probable case. To say, however, that it is constantly enlarged as Dr. Dreschfeld[3] does, appears to be a mistake. In a recent case at the Bristol Royal Infirmary, the spleen weighed only 2 oz., and that in spite of the fact that typhoid bacilli were found in it. It may be further mentioned that of eighteen cases in the *post-mortem* room during recent years, in one the weight was only 4¾ oz., and in two only 6 oz. In none of these could the spleen have been felt during life.

To return to complications: since the blood is deranged, the heart must become an object of attention. The frequency of cardiac enfeeblement in typhoid fever is familiar to all, yet curiously enough the heart rarely suffers in the way seen in some diseases of shorter duration—diphtheria, for example. Sudden death may, however, in rare instances, occur. Osler[4] records such a case, and Sir William Broadbent[5] incidentally mentions the possibility of this event in his Lumleian lectures on prognosis in heart disease. Another interesting case is recorded by Clemow,[6] where, in a man aged 25, dyspnœa and high temperature were found to be associated with dilatation of the heart. Venesection was performed, and the patient recovered. Apparently, in rare

[1] Hawkins, *Lancet*, 1892, ii. 1387.
[2] *Johns Hopkins Hosp. Rep.*, 1895, iv. 83.
[3] Clifford Allbutt's *System of Medicine*, 1896, vol. i., p. 824.
[4] *Johns Hopkins Hosp. Rep.*, 1895, v. 461. [5] *Lancet*, 1891, i. 753.
[6] *Ibid.*, 1896, i. 1495.

instances, fatal syncope may occur, as in diphtheria, after the patient has been deemed well enough to leave his bed. Two or three years ago I read the record of such a case, but regret that I have been unable to find the reference. In these cases of sudden death, it is the heart muscle that has suffered; but one would expect the valves to occasionally suffer also in a disease in which septic organisms are circulating in the blood. Malignant endocarditis[1] does occur, but it occurs rarely; yet, as Osler observes, it is remarkable that it is not present more often. Yet not only septic micro-organisms, but the typhoid bacillus, may give rise to acute endocarditis. Flexner[2] mentions three observers who have found the bacillus in this complication, and Kanthack[3] speaks also of having found the bacillus in the blood of a case of ulcerative endocarditis occurring in typhoid fever, though he does not mention that the bacillus was found in the vegetations.

More important from the point of view of frequency are lesions of the **lungs**, though as a serious complication they cannot be said to be common. Yet in an analysis of 95 fatal cases of typhoid fever by Norman Moore[4] death is attributed to pneumonia in six instances. Consolidation of the lungs may occur in the patchy form of broncho-pneumonia, or as consolidation of a whole lobe not differing in aspect from the red hepatization of ordinary lobar pneumonia. Examples of both have occurred in the *post-mortem* room of the Bristol Royal Infirmary during the recent epidemic, and examination of the past records of 58 cases shows five other cases of lobar pneumonia and three of broncho-pneumonia. The question naturally arises, What is the immediate cause of the pneumonia? In a paper upon secondary infective lesions in lobar pneumonia by Lance and Kanthack,[5] one of the writers doubts whether the typhoid bacillus is capable of giving rise to this complication, and states that in four cases of lobar pneumonia occurring in typhoid fever he has found the pneumococcus. In some instances, therefore, typhoid fever seems to predispose the patient to infection by the pneumococcus. In such a case infection would take place presumably by the air. In broncho-pneumonia as a complication the same may occur. Mills Pearce[6] records five cases of broncho-pneumonia occurring in typhoid fever, and here also the pneumococcus was found in two cases, once alone and once combined with streptococci and staphylococci. In two others the colon bacillus was found alone, and in the fifth, which was complicated with diphtheria, the bacillus diphtheria and staphylococcus aureus were present. The cases above mentioned that occurred recently at the Bristol

[1] Osler, *Johns Hopkins Hosp. Rep.*, 1895, v. 465; Shattuck, *Boston M. & S. J.*, 1892, cxxvii. 277.
[2] *Johns Hopkins Hosp. Rep.*, 1895, v. 352.
[3] *J. Laryngol.*, 1896, x. 178.
[4] *Tr. Path. Soc. Lond.*, 1882, xxxiii. 155. [5] *St. Barth. Hosp. Rep.*, 1897, xxxii. 319.
[6] *Boston M. & S. J.*, 1897, cxxxvii. 563.

Royal Infirmary were, one of the lobar, the other of the lobular form of consolidation, but in both of these Dr. Symes found the bacillus coli. In these cases in which the colon bacillus is found infection of course takes place through the blood, not through the air as in the case of the pneumococcus. One would expect inflammation set up in the lungs by these septic organisms to be more liable to break down than that set up by the pneumococcus, which we know almost invariably ends in resolution if the patient lives. The case of broncho-pneumonia that occurred at the Bristol Royal Infirmary, which was set up, as we have mentioned, by the bacillus coli, showed commencing suppuration, but records of serious lung lesions following typhoid fever do not appear to be common. A case of gangrene of the lung is recorded by Macdonald[1] and another by Acker;[2] and Hale White,[3] in bringing forward two cases of pneumothorax after typhoid fever, agrees with Sir Dyce Duckworth that this complication may sometimes possibly arise from the bursting of a small abscess of the lung into the pleural cavity. No doubt if carefully looked for small abscesses would be found not uncommon.

We have seen that septic micro-organisms may set up inflammation of the lungs. Their power for harm is however not limited to this region of the respiratory system, since the **larynx** also may be attacked. Possibly in some instances expectoration from the diseased area of the lung may convey micro-organisms in an exceptionally virulent condition, and deposit them upon the part that eventually ulcerates. In this connection it may be worth mentioning that ulceration of both vocal cords was present in one of the cases of pneumonia complicating typhoid fever mentioned above that occurred recently at the Bristol Royal Infirmary. In other cases, as Kanthack suggests, the ulceration may be owing to the fact that the lowered vitality of the tissues allows septic micro-organisms, always present in the larynx, to attack the mucous membrane. Ulceration of the larynx is not very infrequent. Kanthack[4] found it mentioned in fourteen of sixty-one reports of autopsies on cases of typhoid fever at St. Bartholomew's Hospital. It is, however, not a serious one, but perichondritis may be superadded, and Parsons[5] records a case in which acute œdema supervened, causing rapid asphyxia.

Another lesion of the throat is inflammation of the **pharynx and tonsils**, sometimes with ulceration. Inflammation in this neighbourhood may predispose to infection of diphtheria, which from time to time attacks typhoid fever patients. Examples of this have occurred during the recent epidemic at the Bristol Hospital for Sick Children, and also at the Bristol General Hospital. Middle-ear disease, when it occurs, may also probably be secondary to a pharyngitis.

[1] *Med. Times & Gaz.*, 1862, ii. 127. [2] *Tr. Am. Pediat. Soc.*, 1896, viii. 141. [3] *Lancet*, 1896, i. 485. [4] *J. Laryngol.*, 1896, x. 174. [5] *Tr. Roy. Acad. M. Ireland*, 1895, xiii. 337.

Although there is little or no evidence that the typhoid bacillus is capable of setting up inflammation in the lungs or air passages, this can hardly be said of the **liver.** The blood, after leaving the infected intestines, reaches, as we know, the liver. There it is deprived of many of the toxic bodies absorbed from the intestinal tract, and in cases of typhoid fever seems to leave behind also typhoid bacilli, which, excreted in the bile, find their way to the gall-bladder. Richardson,[1] who has examined the bile from the gall-bladder in three consecutive cases of typhoid fever for typhoid bacilli and found them in every instance, quotes Chiari as finding them in nineteen out of twenty-two cases. Councilman[2] also says that in his experience the typhoid bacillus may be found in nearly every case, and that he has come to regard the gall-bladder as one of the surest places in which to find this micro-organism. The bacillus in this situation is not harmless. Chiari found evidence of inflammation in thirteen of the nineteen cases in which he discovered the micro-organism in the gall-bladder. In these cases the inflammation presumably was not serious, but it may go on to suppuration, and even lead to perforation of the gall-bladder. A recent record of such a case is given by Hawkins.[3] Monier-Williams and Marmaduke Sheild[4] have recorded a case of peritonitis following perforation of the gall-bladder successfully treated by operation, and another interesting case, in which the distended gall-bladder was recognised clinically, and sero-purulent fluid withdrawn by an aspirator, with recovery of the patient, is recorded by Mason.[5] A rarer complication occurring in the liver is solitary abscess. Osler[6] quotes Hölscher as finding only twelve cases in 2000 *post-mortem* examinations on cases of typhoid fever, while Dopfer found only a slightly higher percentage. In his statistics there were ten cases in 927 autopsies. One case occurred in the *post-mortem* room of the Bristol Royal Infirmary this summer. A still rarer complication than abscess appears to be suppurative pylephlebitis.

The typhoid bacilli not only find their way out of the blood into the bile, but, as is well known, into the urine. In the gall-bladder they are capable of setting up inflammation, and one would expect therefore that in some instances similar trouble should follow in the urinary tract. Workers in the *post-mortem* room are familiar with the cases of pyelitis not due to inflammation ascending from the bladder, but doubtless set up by septic micro-organisms passing out from the blood with the urine, and could in no way be surprised by the interesting observation made at the Johns Hopkins Hospital that in sixty consecutive cases of typhoid fever, no less than ten had pyuria. The typhoid bacillus, however, does not appear to have been responsible for the pus in the urine in many of these.

[1] *Boston M. & S. J.*, 1897, cxxxvii. 570.
[2] *Tr. Ass. Am. Physicians*, 1897, xii. 41. [3] *Lancet*, 1897, i. 313. [4] *Ibid.*, 1895, i. 534
[5] *Tr. Ass. Am. Physicians*, 1897, xii. 23. [6] *Ibid.*, 383.

The typhoid bacillus was found in pure culture once only, and once in combination with the colon bacillus. With one exception the bacillus coli was found in the remaining cases.[1] More serious lesions in the urinary tract may, however, occur, and apparently be set up by the typhoid bacillus. In two cases of abscesses in the kidney occurring in the same hospital Flexner[2] found this bacillus. Although the frequency of pyuria may surprise some of us, the common presence of albuminuria is no new fact. A valuable paper upon this feature emanating from the workers in the same admirable hospital may, however, be referred to.[3] In 164 of 229 cases of typhoid fever, albuminuria was noted, and in 21 there were evidences of a definite nephritis. Of these 7 died, "but in no instance did the renal condition seem to be directly responsible."

Of **parotitis, subcutaneous abscesses, periostitis, osteomyelitis,** and **arthritis** I cannot speak. The numerous complications affecting the **nervous system** in typhoid fever also form far too large a subject to be even briefly touched upon; yet I can hardly close without a reference to meningitis. The occasional marked prominence of cerebral symptoms in typhoid fever is well known, as well as the difficulty sometimes experienced in diagnosing the case from one of meningitis; and I have to confess that, under such circumstances, I have fallen into error. Although marked cerebral symptoms may have been present, on the *post-mortem* table, as a rule, the brain to the naked eye shows nothing abnormal. Yet meningitis may be found in rare instances, and in connection with the etiology of this inflammation, it is worthy of note that Flexner[4] states that in no less than seven cases of cerebro-spinal meningitis occurring in typhoid fever the typhoid bacillus has been found.

THEODORE FISHER.

### SURGERY.

[1] Blumer, *Johns Hopkins Hosp. Rep.*, 1895, v. 327.

[2] *Ibid.*, 343.

[3] Hewetson, *Ibid.*, 1895, iv. 113.

[4] *Ibid.*, 1895, v. 352.

